# Neighborhood Poverty in Combination with Older Housing Is Associated with Adverse Birth Outcomes: A Study on Ubiquitous Lead Risk among 1 Million Births in Texas

**DOI:** 10.3390/ijerph19031578

**Published:** 2022-01-29

**Authors:** Bethany Marie Wood, Catherine Cubbin

**Affiliations:** The Steve Hicks School of Social Work, University of Texas, Austin, TX 78712, USA; ccubbin@austin.utexas.edu

**Keywords:** census tract, housing age, neighborhood poverty, birth outcomes, preterm birth, low birth weight, small-for-gestational age, ubiquitous lead

## Abstract

The purpose of this study was to determine whether housing age in combination with neighborhood poverty, as a proxy for fetal exposure to heavy metal lead, is associated with adverse birth outcomes. We linked population-level birth certificate data for Black, Hispanic, White and Other women, stratified by nativity, from 2009–2011 in Texas (*n* = 1,040,642) to census the tract-level median housing age/poverty level from the American Community Survey, 2007–2011. Tracts with median housing age values before 1975 with a poverty level of 20% or more were considered to be neighborhoods with a high risk of exposure to deteriorating lead-based paint. We estimated multilevel models to examine the relationship between neighborhood housing age/poverty level and each dependent variable (preterm birth, low birth weight, small-for-gestational age). The odds of adverse birth outcomes were significantly higher for mothers living in high-poverty neighborhoods with median housing built before the lead-based paint ban. Increased awareness of—and improved methods of alleviating— ubiquitous lead-based paint exposure in Texas may be necessary interventions for positive developmental trajectories of children. Allocating federal funds for place-based interventions, including universal lead paint mitigation, in older, high-poverty neighborhoods may reduce the disproportionate risk of adverse birth outcomes.

## 1. Introduction

Negative birth outcomes in the United States of America are common and consequential. In 2018, 1 out of every 10 infants was born preterm (less than 37 weeks), and 1 in 12 exhibited low birth weights [1]. Early and low birth weights have been linked to a number of harmful states in the later lives of children, such as altered cardiovascular and kidney function, increased risks of social and psychological problems, and higher odds of a diagnosis of cerebral palsy, and early births are the leading cause of perinatal mortality [2,3,4]. In recent years, Texas performed near the national average at producing healthy babies, with a preterm birth rate of 11% in 2018 and a low-birth-weight rate of 8.3% in 2017 [1,5]. For its similarity to the U.S.A. as a whole, as well as its large size both in land and population, we use geographic data on Texas that may be representative of the U.S.A. in order to investigate a potential trigger for harmful birth outcomes.

Researchers have investigated a number of potential causes for negative birth outcomes. Maternal infection, hypertension, pre-eclampsia, maternal stress, and multiple pregnancies are significant predictors of preterm birth and low birth weight, though this list is non-exhaustive [6,7]. Of interest to this study, fetal exposure to the heavy metal lead (Pb), a known toxicant, precipitates several adverse birth outcomes, including preterm labor and small-for-gestational-age births [8,9].

Inhalation of lead from leaded gasoline was the primary source of elevated blood lead levels in the past, but exposure via gasoline plummeted after the U.S. ban on leaded gasoline [10]. Currently, the main source of ubiquitous lead exposure is through inhalation or ingestion of lead-based paint and dust contaminated by lead in housing, followed by the presence of Pb in contaminated water [2]. Housing age is a well-studied physical determinant of health [11] and is often tied to the geographic location of the houses in that neighborhoods tend to have houses built at similar times [12]. Moreover, low-income neighborhoods are often those with older homes [13]. Before the mid-1970s, when the Consumer Product Safety Commission was created, most houses in the U.S.A. were painted with leaded paint [14], because it accelerated drying and was durable. As a result, many of the dwellings standing today that were built before 1978 still contain some amount of leaded paint, which can be harmful in deteriorating housing that produces lead dust and paint chips [15,16]. Furthermore, low-income neighborhoods also face higher exposure to Pb in water [17].

Although there is an indistinct relationship between many environmental metals and adverse birth outcomes, that is not the case for the relationship between lead exposure and adverse birth outcomes [18,19,20]. The risks of adverse birth outcomes are greater with early-pregnancy lead exposure. Cantonwine et al. [21] found that lead exposure during the first and second trimesters was associated with the greatest risk of premature delivery. However, other studies have found that high lead levels in umbilical cord/maternal blood at the time of birth was associated with risk of premature births, suggesting that exposure to lead late in pregnancy may also be harmful [22].

Despite the relationships between ubiquitous lead exposure and adverse birth outcomes, studies looking at lead exposure from house paint have become less common since the ban on lead-based paint in the mid-1970s [18]. However, the lead in house paints is still a prevalent health concern that should not be overlooked [23], given that many low-income communities have homes built before the lead-paint ban that may continue to affect birth outcomes through their deterioration over time [13]. The emergence of higher blood-lead levels for young children in rural areas compared to those in urban areas is additionally a recent and concerning trend [24], suggesting that there may be different lead exposure pathways based on rurality or urbanization. The population in 136 (over 50%) of the 254 counties in Texas are considered majority rural [25], which perhaps makes Texas a uniquely important state for examining communities at risk for lead exposure in rural compared to urban areas. The risk of ubiquitous lead exposure is especially concentrated in economically disadvantaged communities of color where the housing age is associated with higher levels of lead in blood [26].

**Hypothesis** **1** **(H1).** *Living in an older, high-poverty neighborhood increases the odds of preterm birth, low birth weight, and small-for-gestational age compared to living in a newer and/or low poverty neighborhood in Texas.*

**Hypothesis** **2** **(H2).** *High-poverty neighborhoods with median housing built before the lead-based paint ban will be concentrated in rural areas.*

**Hypothesis** **3** **(H3).** *The urban/rural status of a census tract will significantly moderate the relationship between at-risk neighborhoods and the odds of preterm birth, low birth weight, and small-for-gestational age.*

**Hypothesis** **4** **(H4).** *There will be racial/ethnic disparities, extenuated by nativity status, in the prevalence of preterm birth, low birth weight, and small-for-gestational age for Black and Hispanic women compared to White women.*

In this study, we use population-level data from Texas to document associations between living in pre-1975 housing and high-poverty neighborhoods and negative birth outcomes. Furthermore, we present geospatial information on these neighborhoods. Our study is the first to link negative birth outcomes in Texas using comprehensive birth certificate data linked to geographic census tract-level housing and poverty data, and to examine racial/ethnic groups by nativity.

## 2. Materials and Methods

We obtained restricted individual-level birth certificate data from natality files for all singleton births to non-Hispanic Black, Hispanic, non-Hispanic Other, and non-Hispanic White women from 2009–2011 in Texas (*n* = 1,128,679) [27]. To arrive at our analytic sample, we first excluded records missing length of gestation or birth weight, those with gestational age < 22 or >44 weeks, and those with implausible combinations of birth weight and gestational age [28]. We further excluded records missing geocodes, resulting in 92.2% of the total singleton births (*n* = 1,040,642).

Census tract-level median housing age data were obtained from the American Community Survey, 2007–2011, and census tract-level poverty data were obtained from the American Community Survey, 2006–2010 [29,30]. Tracts with median housing age values before 1975 with high poverty levels (≥20%) were considered to be environments that were highly likely to contain lead-based paint in addition to lead from other sources, such as plumbing or soil [2,31]. Economic Research Service (ERS) Rural–Urban Continuum Codes were obtained from the U.S. Department of Agriculture, 2013 [32]. Counties’ Rural–Urban Continuum Codes were matched to census tracts within their corresponding counties.

We linked individual-level birth certificate data to census tract-level data and ERS Rural–Urban Continuum Codes based on residential geocodes, provided with the birth certificate data from the Department of State Health Services. These mothers lived in 99.0% of census tracts in Texas (5208 out of a total of 5265), with an average of 200 mothers per tract (range = 1–1873). Nearly 97% of the census tracts had at least 30 births, indicating substantial clustering by census tract.

### 2.1. Individual Measures

Our dependent variables were preterm birth, low birth weight, and small-for-gestational age. The three dependent variables were each binary coded into 0 and 1, with 1 indicating the presence of preterm birth, low birth weight, or small-for-gestational age. Preterm birth was defined as fewer than 37 weeks gestation, low birth weight was defined as <2500 g, and small-for-gestational age was defined as <10th percentile of birth weight-for-gestational age using published national reference guidelines [33]. Other individual measures included the child’s sex (female, male), maternal age at delivery (11–19 years, 20–34 years, 35 years or older), maternal marital status (unmarried, married), maternal parity (first birth, 2nd–4th birth, ≥5th birth), maternal education level (less than high school, high school or graduate equivalency diploma, some college, college graduate), timing of prenatal care (first trimester care, no first trimester care), and maternal race/ethnicity/nativity (non-Hispanic Black, Hispanic, non-Hispanic White, non-Hispanic Other, each stratified by nativity [U.S.-born, immigrant]). Given documented racial/ethnic disparities in adverse birth outcomes in Texas [34], U.S.-born non-Hispanic White women were chosen as the reference category, given that they are the most socially advantaged racial/ethnic group. The father’s education level was also included (missing, less than high school, high school or graduate equivalency diploma, some college, college graduate). All variables came from birth certificate records and had few missing data (<5%) except for the father’s education (14.6%); thus, we included a category for missing for that one variable.

### 2.2. Neighborhood Measures

The median housing age at the tract level was first coded into a dichotomous variable, with 1 as a median age before 1975 and 0 as a median age of 1975 or after. Next, census tract poverty level, representing the percentage of census tract residents living below the federal poverty line, was divided using the American Community Survey cutoff score for census tracts designated as “poverty areas” (≥20% of the tract population lives below the federal poverty line) [35]. Then, the final “at-risk neighborhood” variable was created to include the poverty level for each tract creating a dichotomous variable, with 1 as a median age before 1975 and a poverty level of 20% or more, and 0 as either a median age of 1975 and after and/or a poverty level less than 20%. The 0 reference category, therefore, included neighborhoods that were both newer and low poverty, or only one of those classifications. Tracts coded as 1 in this final variable were considered “at-risk neighborhoods” and tracts coded as 0 were considered “not-at-risk neighborhoods”. As few tracts were missing housing age or poverty level data (e.g., industrial areas), this final variable included 98.0% of the tracts in Texas (5138 out of 5265).

Urban and rural status for neighborhood tracts was created from the ERS Rural–Urban Continuum Codes for counties across Texas. The county codes are available in three metropolitan groupings (1—counties in metro areas of 1 million population or more, 2—counties in metro areas of 250,000 to 1 million population, 3—counties in metro areas of fewer than 250,000 population) and six non-metropolitan groupings (4—urban population of 20,000 or more, not adjacent to a metro area; 5—urban population of 20,000 or more, adjacent to a metro area; 6—urban population of 2500 to 19,999, adjacent to a metro area; 7—urban population of 2500 to 19,999, not adjacent to a metro area; 8—completely rural or less than 2500 urban population, adjacent to a metro area; and 9—completely rural or less than 2500 urban population, not adjacent to a metro area), resulting in a nine-part county classification. Census tracts were matched with their corresponding counties and then the codes were dichotomized into metropolitan and non-metropolitan based on the two metropolitan and non-metropolitan groupings provided by ERS. Tracts coded as 1 were considered urban (comprised of the three metropolitan codes) and tracts coded as 0 were considered rural (comprised of the six non-metropolitan codes).

### 2.3. Analyses

The pseudo intra-class correlations (ICC) were 1.1–1.5%, depending on the birth outcome, and the variances between neighborhoods were all significant (τ_00_ = 0.04 to 0.05, *p* < 0.0001). Although the ICCs are low, high clustering and significant variance between neighborhoods implied that our decision to use multilevel modeling was appropriate. The confidence level was set at 95% (*p* < 0.05) to determine the statistical significance. We first examined the distribution of the variables and prevalence of the dependent variables (preterm birth, low birth weight, small-for-gestational age) overall and by individual-level characteristics and neighborhood-level housing age with and without poverty level. Next, we examined preterm birth stratified by housing age/poverty level and race/ethnicity/nativity. Finally, we estimated three sets of hierarchical generalized linear models (HGLM) to examine the relationship between neighborhood housing age and each dependent variable. Multilevel models were built sequentially: (a) bivariate models, (b) a demographic model, (c) a full model, and (d) an interaction model. The HGLM was estimated using Laplace based on a binomial distribution and logit link function which allowed us to assess model fit by examining the change in the negative two log-likelihood (-2LL) between models and comparing AIC and BIC values [36,37]. We used ArcGIS to map out at-risk neighborhood status for census tracts and SAS software version 9.4 for all other analyses.

## 3. Results

Approximately 49% of the births were female, 75% of mothers were between the ages of 20 and 34, and 49% of births were to Hispanic mothers (including immigrant and U.S.-born). Stratified by nativity status, about 33% of births were to U.S.-born White mothers and about 10% were to U.S.-born Black mothers. There were very few births to immigrant Black or White women and 80% of births to “Other” race/ethnicity were immigrants (and likely to be mostly women of Asian descent given Texas demographics). About 58% of the births were to unmarried women, and about 40% were first births. About 52% of the women had a high school degree or less education, and approximately 22% were college graduates. About 15% of the births were missing father’s education, and about 46% of the fathers had a high school degree or less education. About 39% of the women started prenatal care after the first trimester (i.e., delayed) or had no prenatal care, 66% of them lived in neighborhoods classified as urban, about 32% of them lived in neighborhoods where the median age of housing was before 1975, about 39% of them lived in neighborhoods where the poverty level was 20% or higher, and about 20% of them lived in neighborhoods where the median age of housing was before 1975 and the poverty level was 20% or higher (Table 1).

Ten percent of births were classified as preterm birth, 7% were low birth weight, and 11% were small-for-gestational age. The prevalence of adverse birth outcomes was highest for 11-to-19-years-old mothers, Black U.S.-born mothers, unmarried mothers, parents who were less educated (or missing information on father’s education), mothers with delayed or no prenatal care, mothers living in rural neighborhoods, mothers living in neighborhoods with older housing, mothers living in neighborhoods with a poverty level of 20% or higher, and mothers living in at-risk neighborhoods (both older housing and a poverty level of 20% or higher). The prevalence of adverse birth outcomes was lowest among White immigrant mothers, married mothers, parents who were college graduates, mothers without delayed/no prenatal care, mothers living in urban neighborhoods, mothers living in neighborhoods with newer housing (1975 or newer), mothers living in neighborhoods with a poverty level less than 20%, and mothers living in neighborhoods with newer housing and/or a poverty level less than 20%. The lowest prevalence of adverse birth outcomes differed among age groups, with preterm birth and low birth weight being lowest for 20-to-34-years-old mothers, and small-for-gestational age being lowest for mothers aged 35 or higher. The prevalence of birth outcomes varied by parity status where preterm birth was most prevalent for mothers with five or more births, whereas low birth weight and small-for-gestational age were most prevalent for mothers with their first births.

**Figure 1:**
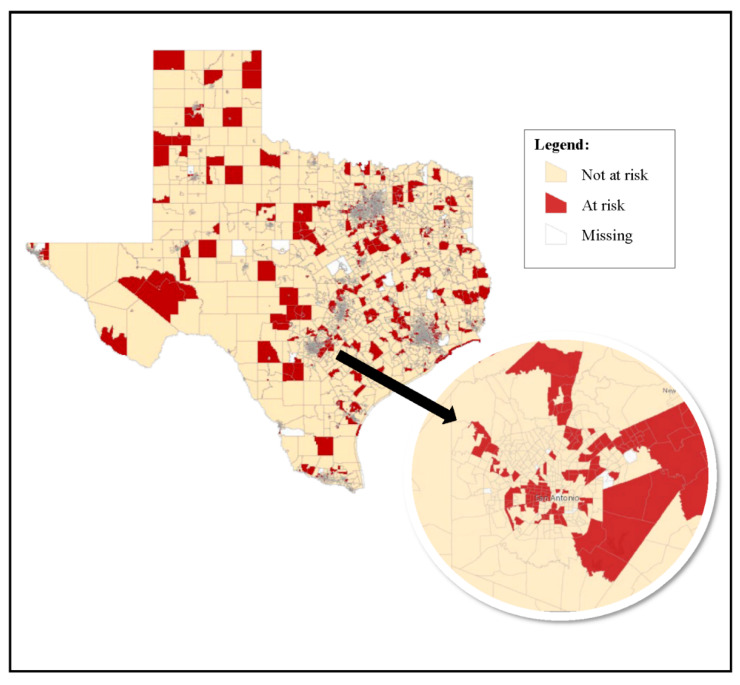
At-risk neighborhoods in Texas, 2010, census tracts, *n* = 5265 Note. At-risk neighborhoods were defined as census tracts with a median housing age < 1975 and a poverty level of ≥20%. Not-at-risk neighborhoods are census tracts with a median housing age of 1975+ and/or a poverty level <20%. Missing indicates census tracts where either median housing age or poverty level were unavailable. Source: American Community Survey, 2007–2011.

Figure 1 displays census tracts by their neighborhood risk status (“at risk” are neighborhoods with older housing and high poverty and “not at risk” are neighborhoods with newer housing and/or lower poverty). At-risk neighborhoods are spread throughout the approximately 270,000 square miles of the state; however, small patterns are visible at the city level. For example, when examining the city of San Antonio, many of the surrounding census tracts have a median housing age older than 1975 and poverty equal to or greater than 20%, indicating a high risk of lead paint and subsequent lead exposure. Additionally, within the city, at-risk neighborhoods are clustered around historically redlined neighborhoods [38,39]; as many of these neighborhoods are still racially concentrated, the risk of lead exposure may be higher for these predominantly Black and Hispanic communities. Similar patterns exist for many of the other major cities and surrounding areas across the state. Furthermore, 87.1% of at-risk neighborhoods were in urban census tracts.

**Figure 2:**
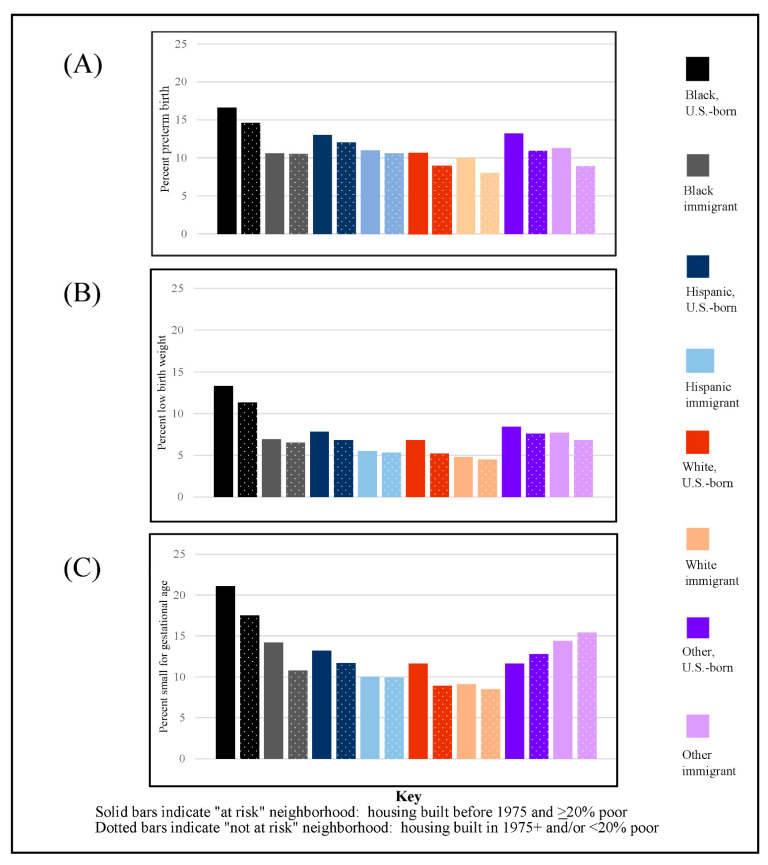
Percent preterm birth (graph **A**), low birth weight (graph **B**), and small-for-gestational age (graph **C**) by race/ethnicity/nativity and risk status, 2009–2011 Texas natality files, *n* = 1,040,642. Sources: American Community Survey, 2007–2011, and birth certificate data from the Texas Department of State Health Services, 2009–2011.

Figure 2 depicts the prevalence of preterm birth, low birth weight, and small-for-gestational age by at-risk neighborhood status and race/ethnicity/nativity. U.S.-born Black mothers in at-risk neighborhoods exhibited a twice higher prevalence of preterm birth compared to White immigrant women living in not-at-risk neighborhoods, translating into one of every six Black women and one out of every twelve White women in those circumstances. Within race/ethnicity, prevalence rates of preterm birth were higher for women living in at-risk versus not-at-risk neighborhoods. Within nativity, U.S.-born women had higher preterm birth prevalence rates than their respective immigrant counterparts, (i.e., U.S.-born Hispanic women had a higher prevalence of preterm birth than immigrant Hispanic women). The findings were similar for low birth weight and small-for-gestational age, with the exception of the “Other” racial category in small-for-gestational age where immigrant women had a higher prevalence compared to U.S.-born women.

Table 2 shows the unadjusted random intercept models for preterm birth, including the odds for at-risk neighborhoods; the random intercept model for at-risk neighborhoods controlling for individual-level demographic characteristics; and, finally, the full model for at-risk neighborhoods, also controlling for socioeconomic characteristics. All variables, with the exception of Other immigrant women, were statistically significant in the unadjusted models, including at-risk neighborhoods. Women who lived in at-risk neighborhoods had 23% higher odds of preterm birth compared with women living in not-at-risk neighborhoods. Those higher odds decreased to 10% after controlling for demographic characteristics, which also remained statistically significant with the same exception of Other immigrant women. In the full model, women who lived in at-risk neighborhoods had 7% higher odds of preterm birth compared with women living in not-at-risk neighborhoods, with additional controls for mother’s and father’s education and timing of prenatal care initiation. In the interaction model, the effect of at-risk neighborhood status on preterm birth was similar in both urban and rural areas and therefore rural/urban status was not a significant moderator. In the final model, all variables remained statistically significant including race/ethnicity/nativity, with U.S.-born Black women having 53% higher odds of preterm birth compared with U.S.-born White women in the fully adjusted model. The full model had the best fit to the data, and 58.83% of the total between-neighborhood variance in preterm birth was explained by it.

Table A1 (see Appendix A) presents models for low birth weight. In the unadjusted model, all variables were significant (except Hispanic immigrants), including women living in at-risk neighborhoods who had about 24% higher odds of delivering a low-birth-weight baby. Those odds were reduced to 14% in the model adjusting for demographic characteristics. After also controlling for mother’s and father’s education and timing of prenatal care initiation, the odds for women living in at-risk neighborhoods were reduced to 9% compared with women living in not-at-risk neighborhoods. In the interaction model, the effect of at-risk neighborhood status on low-birth-weight birth was similar in both urban and rural areas; therefore, rural/urban status was not a significant moderator. In the final model, all variables remained statistically significant including U.S.-born Black women having over two-fold increased odds of low birth weight compared with U.S.-born White women. The full model had the best fit to the data, and 74.5% of the total between neighborhood variance in low birth weight was explained by it.

Table A2 (see Appendix A) presents models for small-for-gestational age. In the unadjusted model, women living in at-risk neighborhoods had 22% higher odds of delivering a small-for-gestational age baby. Those odds were reduced to 11% in the model, adjusting for demographic characteristics. After also controlling for mother’s and father’s education and timing of prenatal care initiation, the odds for women living in at-risk neighborhoods were reduced to 7% compared with women living in not-at-risk neighborhoods. In the first bivariate models, all variables were significant. In the demographic model, White immigrant status was non-significant, and in the final model, women ages 35 and over, and immigrant White status was non-significant. All other variables remained statistically significant including the other categories of race/ethnicity/nativity, with U.S.-born Black women having nearly two-fold increased odds of small-for-gestational age compared with U.S.-born White women. In the interaction model, the effect of at-risk neighborhood status on small-for-gestational age was similar in both urban and rural areas; therefore, rural/urban status was not a significant moderator. The full model had the best fit to the data, and 70.5% of the total between neighborhood variance in small-for-gestational age was explained by it.

## 4. Discussion

In support of the primary hypothesis, the odds of giving birth to a preterm, low-birth-weight, or small-for-gestational-age infant were all significantly higher for Texas mothers living in areas with greater concentrations of housing built before the ban on lead-based paint with high neighborhood poverty from 2009 to 2011. Individual-level sociodemographic characteristics explained much of the variation in the odds of experiencing negative birth outcomes, especially mothers’ race/ethnicity/nativity, but could not fully diminish the association between living in an at-risk neighborhood and inauspicious births.

Many studies document the relationship between heavy metal lead and increased risk of adverse birth outcomes [18,19,20,21,22]. However, previous studies taking an ecological approach have also found that the risk of environmental exposure to lead is associated with harmful health outcomes for children, including several recent studies using a similar independent variable (housing age and neighborhood poverty) reporting that neighborhood-level risk of lead exposure was associated with poor cognitive performance for children [40,41,42,43]. Furthermore, more studies have confirmed that housing age and neighborhood poverty are correlated with childhood exposure to lead [44,45]. This study significantly contributes to the literature as it is the first to use an ecological lens to examine neighborhood-level risk of lead exposure on all birth outcomes in Texas. While the neighborhood-level measure of risk of exposure to lead cannot be applied to all individuals in a given neighborhood (per the ecological fallacy), overlooking the effects of context and place when it comes to health outcomes (also known as the atomistic fallacy) may miss significant critical structural inequalities that impact health [46]. Adding to the novelty of this study, the findings are particularly salient given that this study is the first to link all Texas birth certificate data to an independent variable that includes both built environmental components of housing age, and the social components of neighborhood poverty level and basing the housing age cutoff around a lead-based paint policy.

The secondary hypothesis, that Texas rural areas would have more at-risk neighborhoods than urban neighborhoods, was not supported by the study results. Instead, across the state of Texas, high-poverty neighborhoods with median housing built before the ban on lead-based paint (at-risk neighborhoods) were concentrated in urban census tracts. This coincides with previous literature on other states. For example, one study in South Carolina found a higher prevalence of blood lead levels in urban areas and that older housing age is a major factor in increasing blood lead levels [24]. In this study, despite Texas having a high number of poor rural areas [25], trends follow those of smaller and less overall rural states. These results demonstrate the need for more data on blood lead levels in these urban at-risk Texas neighborhoods.

The third hypothesis, that the urban/rural status of a census tract would moderate the relationship between at-risk neighborhoods and the odds of the adverse birth outcomes, was not supported. These study findings may indicate that older, high-poverty neighborhoods have higher risk regardless of urban/rural status, or perhaps that other structural patterns exist. The geospatial analysis of at-risk neighborhoods often matched maps of historically redlined neighborhoods in major Texas cities. Given that historical redlining is associated with higher rates of preterm birth in cities such as New York City [47], the potential impact of structural racism in this study cannot be ignored.

In support of the final hypothesis, U.S.-born Black mothers had the highest risk for all of the adverse birth outcomes compared to U.S.-born White mothers, and U.S.-born Hispanic women also had higher risk compared to U.S.-born White mothers; these findings add support to a growing body of literature that highlights structural racism as a key determinant in birth outcomes [48]. Racial discrimination [49] and structural racism at the neighborhood level are associated with increased adverse birth outcomes [50]. This study contributes to this body of literature further by examining nativity. The higher risk by nativity status for U.S.-born Black and Hispanic mothers may also support the impact of structural racism as a factor impacting health across the lifespan, as immigrant Black and Hispanic women had less risk. In other words, experiencing structural racism since early childhood may have greater effects on adult pregnancy and birth outcomes, as demonstrated by the higher prevalence of adverse birth outcomes for U.S.-born Black and Hispanic mothers compared to their immigrant counterparts. This finding matches existing literature on the “immigrant paradox” for birth outcomes where immigrant women of color have less risk of adverse birth outcomes compared to their U.S.-born counterparts [51]. Furthermore, the racial/ethnic/nativity disparities in this study add support that structural racism and discrimination continue to affect health outcomes, and risk of lead exposure is perhaps a resulting manifestation of structural racism, along with stress, that directly and indirectly impacts adverse birth outcomes.

Since a large portion (19.4%, or about 200,000 mothers) of the population resided in at-risk neighborhoods, the primary implication is support for place-based interventions that reduce poverty and inequalities in the built environment, particularly for high poverty neighborhoods with median housing age built before the ban on lead-based paint. First, further examination of environmental factors, including the presence of lead-based paint, lead-contaminated water, pollution, and other known correlates of adverse birth outcomes can identify the best points of intervention for the individual at-risk neighborhoods [17,18,52]. Once points of intervention are identified in each respective neighborhood, funding should be allocated for revitalization efforts that address the most detrimental environmental factors through place-based interventions.

Rehabilitation efforts have been implemented across cities in the U.S.A. to decrease exposure to lead [53]. For example, studies of place-based interventions and decades of rehabilitation efforts in Butte, Montana presented promising results [54]. Following testing in Butte neighborhoods, housing age was found as one of the strongest predictors of paint lead, soil lead, and dust lead concentrations, but importantly, only house dust lead (from lead-based paint) was directly related to blood lead. Decades of rehabilitation efforts were then targeted at reducing residential metals, and a recent study presented significant declines in blood-lead levels across Butte since these place-based interventions took place [54]. Given that the median housing age before the ban on lead-based paint in high-poverty neighborhoods significantly predicted adverse birth outcomes in Texas, interventions that ameliorate deteriorating housing conditions in these neighborhoods may prevent future adverse birth outcomes.

The racial/ethnic/nativity disparities in adverse birth outcomes and the overlap of urban at-risk neighborhoods with historically redlined neighborhoods may indicate that funding for place-based interventions should especially be allocated to older, high-poverty communities of color. In Texas, addressing structural racism in low-income historically redlined neighborhoods may mean allocating funding to mitigate existing environmental injustices linked to adverse birth outcomes, including air pollution, and lead-contaminated water [55]. Furthermore, as structural racism and discrimination are linked to increased maternal stress, a known predictor of adverse birth outcomes [6,7], Texas health professionals should be informed that U.S.-born Black and Hispanic mothers from older, high-poverty neighborhoods face the highest risk of adverse birth outcomes. Additionally, increased access to child healthcare for children already impacted by these adverse birth outcomes in older, high-poverty neighborhoods is needed.

Other pathways, such as risks associated with structural racism (i.e., pollution) [52], may also account for the findings in this study, which is why place-based interventions are identified as the first and primary implication. As this study only measured the risk of exposure to lead-based paint, further testing of lead-based paint in these at-risk neighborhoods is necessary to identify houses with lead-based paint or other sources of ubiquitous lead in these communities.

### 4.1. Testing and Addressing Ubiquitous Lead Risk

Currently, the regulatory body in charge of monitoring lead levels in the U.S.A. is the Environmental Protection Agency, which directs its lead-monitoring and enforcement efforts through its office of Enforcement and Compliance Assurance. Most of the strength of the agency and office is exercised through the Lead Renovation, Repair, and Painting rule (LRRP) [56], which regulates the handling of known lead paint in older buildings during renovation, repair, or painting in order to assure that existing lead paint is minimally disturbed and to limit the exposure of people to lead dust. Other rules mandate that the owners of older buildings must disclose known lead paint on their premises to potential residents. Actions that the Office of Enforcement and Compliance Assurance (OECA) can take to combat the effects of lead exposure mostly take the form of litigation support for aggrieved parties once lead exposure has already occurred. Additionally, there may be important geographic methods to prevent future lead exposure using tools such as the Environmental Lead Index [57].

While notifying potential residents of the presence of lead and regulating building renovations may lessen some lead exposure, these sources of mitigation will mostly benefit individuals and families who possess the resources to have choices when deciding where to live, and those who can afford to live in renovated properties. Ideally, federal funds would be set aside to institute a universal lead paint testing program for older buildings and to properly contain the lead in affected buildings.

### 4.2. Limitations

This study is limited by short-term, cross-sectional, census-tract level housing and poverty data, as well as a limited set of socioeconomic factors. Longitudinal studies using individual-level housing characteristic indicators are needed to obtain more precise estimates of the association between living in pre-lead-ban housing and adverse birth outcomes. Despite these limitations, this study is bolstered by a large, population-level sample of over 1 million births; utilization of all eligible birth certificate data in Texas; comparison by nativity for Hispanic, Black, and White mother subgroups; objective measures of birth outcomes from the birth certificate data; objective rather than subjective median housing age and poverty level for census tracts; an appropriate multilevel modeling strategy; and fairly recent data.

## 5. Conclusions

This study adds to the evidence that housing age in combination with neighborhood poverty is still a significant predictor of adverse birth outcomes; areas with older median housing ages and high poverty appear to be most at risk of these birth disparities. More broadly, findings identify older housing and high-poverty neighborhoods as potential structural determinants of adverse birth outcomes. Additionally, structural racism likely plays an important factor as U.S.-born Black and Hispanic women face disproportionate odds of these adverse birth outcomes compared to U.S.-born White women. Furthermore, poor families, families living in high-poverty neighborhoods, residents in older neighborhoods within urban or historically segregated areas, U.S.-born Black and Hispanic mothers, and those subject to other choice-limiting vulnerabilities will likely continue to face disproportionate rates of adverse birth outcomes without place-based interventions.

This study points to the need for increased awareness of the prevalence of lead-based paint in older, high-poverty neighborhoods in Texas and broadly across the U.S.A. Despite the ban on lead-based paint, the measure of risk of exposure to lead was associated with increased adverse birth outcomes. Integrated surveillance of housing characteristics, neighborhood poverty, sociodemographic characteristics of mothers, and birth outcomes will be critical for future investigations of whether living in low-income neighborhoods and older housing plays a lasting role in children’s developmental trajectories.

## Figures and Tables

**Table 1:** Descriptive characteristics, 2009–2011 Texas natality files, *n* = 1,040,642.

Characteristic	% Distribution	% Preterm Birth	% Low Birth Weight	% Small-for-Gestational Age
Total		10.4	6.8	11.3
Child’s sex				
Female	48.9	10.4	7	13.7
Male	51.1	11.5	6.1	9.1
Mother’s age (range 11–57)				
11–19	12.5	12.8	8.3	15.3
20–34	75.3	10.3	6.2	11
35+	12.2	12.6	7	9.5
Mother’s race/ethnicity				
Black, U.S.-born	10.3	15.1	11.8	18.4
Black, immigrant	1.1	10.5	6.5	11.1
Hispanic, U.S.-born	26.8	12.3	7.1	12.1
Hispanic, immigrant	22.5	10.7	5.4	9.9
White, U.S.-born	32.6	9	5.3	9.2
White, immigrant	1.7	8.2	4.5	8.6
Other, U.S.-born	1	11.2	7.6	12.7
Other, immigrant	4	9	6.9	15.4
Mother’s marital status				
Unmarried	42.5	12.5	7.9	13.7
Married	57.5	9.7	5.5	9.6
Parity				
First birth	39.6	10.5	7.7	13.9
Second–fourth birth	56	10.9	5.7	9.7
Fifth+ birth	4.4	15.4	7.5	10.1
Mother’s education				
Did not finish high school	25.4	12.8	7.3	12.8
High school graduate	27	11.6	7.2	12.6
Some college	26.2	10.7	6.5	11
College graduate	21.5	8.1	4.9	8.7
Father’s education				
Missing	14.6	18.8	9.2	15.3
Did not finish high school	20.9	11.8	6.6	11.5
High school graduate	24.7	11.3	6.9	11.9
Some college	21	10.2	5.9	10.2
College graduate	18.9	7.9	4.7	8.8
Delayed/no prenatal care				
Yes	39.3	10.8	7	13.2
No	60.7	10.7	6.2	10.2
Urban/rural status				
Urban	66	10.9	6.5	11.3
Rural	34.1	11.7	6.9	12.2
Median Housing age				
<1975	32.2	11.8	7.2	12.3
1975+	67.7	10.6	6.2	10.9
Poverty level				
≥20% poor	38.5	12.3	7.3	12.5
<20% poor	61.5	10.1	6.1	10.6
Median Housing age + poverty level				
<1975 and ≥20% poor	19.4	12.5	7.7	13
1975+ and/or <20% poor	80.6	10.6	6.3	11

Sources: American Community Survey, 2007–2011, U.S. Department of Agriculture Rural Urban Continuum Codes, 2013, and birth certificate data from the Texas Department of State Health Services, 2009–2011.

**Table 2:** Odds and 95% confidence intervals of preterm birth, 2009–2011 Texas natality files, *n* = 1,040,642.

Characteristic	Bivariate Models	Demographic Model	Full Model
Child’s sex			
Female	0.89 (0.88–0.90)	0.89 (0.88–0.90)	0.89 (0.88–0.90)
Male	1	1	1
Mother’s age (range 11–57)			
11–19	1.22 (1.20–1.24)	1.12 (1.10–1.15)	1.03 (1.01–1.06)
20–34	1	1	1
35+	1.30 (1.27–1.32)	1.32 (1.30–1.35)	1.38 (1.35–1.41)
Mother’s race/ethnicity/nativity			
Black, U.S.-born	1.75 (1.72–1.79)	1.61 (1.58–1.65)	1.53 (1.49–1.56)
Black, immigrant	1.18 (1.12–1.26)	1.16 (1.08–1.23)	1.16 (1.09–1.24)
Hispanic, U.S.-born	1.33 (1.31–1.36)	1.25 (1.23–1.28)	1.17 (1.15–1.19)
Hispanic, immigrant	1.16 (1.13–1.18)	1.08 (1.06–1.10)	0.95 (0.93–0.97)
White, U.S.-born	1	1	1
White, immigrant	0.91 (0.86–0.96)	0.90 (0.85–0.96)	0.93 (0.88–0.99)
Other, U.S.-born	1.27 (1.20–1.35)	1.27 (1.19–1.35)	1.26 (1.18–1.35)
Other, immigrant	1.01 (0.97–1.05)	1.03 (0.99–1.07)	1.07 (1.03–1.11)
Mother’s marital status			
Unmarried	1.28 (1.27–1.30)	1.20 (1.18–1.22)	1.09 (1.07–1.11)
Married	1	1	1
Parity			
First birth	1	1	1
Second–Fourth birth	1.03 (1.01–1.04)	1.07 (1.05–1.08)	1.03 (1.01–1.05)
Fifth+ birth	1.49 (1.44–1.53)	1.45 (1.40–1.49)	1.32 (1.28–1.36)
Mother’s education			
Did not finish high school	1.58 (1.55–1.62)		1.34 (1.31–1.38)
High school graduate	1.42 (1.39–1.45)		1.20 (1.17–1.23)
Some college	1.31 (1.28–1.34)		1.14 (1.11–1.17)
College graduate	1		1
Father’s education			
Missing	1.85 (1.81–1.89)		1.43 (1.39–1.48)
Did not finish high school	1.51 (1.48–1.55)		1.26 (1.22–1.29)
High school graduate	1.45 (1.41–1.48)		1.24 (1.20–1.27)
Some college	1.30 (1.27–1.33)		1.17 (1.14–1.21)
College graduate	1		1
Delayed prenatal care			
Yes	0.98 (0.97–0.99)		0.88 (0.87–0.89)
No	1		1
Urban/rural status			
Urban	0.93 (0.91–0.96)	0.92 (0.90–0.95)	0.96 (0.93–0.98)
Rural	1	1	1
Median housing age + high poverty, tract level			
<1975 & ≥20% poor	1.23 (1.20–1.25)	1.10 (1.08–1.12)	1.07 (1.04–1.09)
1975+ and/or <20% poor	1	1	1
U_0j_		0.026	0.02
-2LL		703,088.00	663,655.70
AIC		703,122.00	663,705.70
BIC		703,233.30	663,869.30

Sources: American Community Survey, 2007–2011, U.S. Department of Agriculture Rural Urban Continuum Codes, 2013, and birth certificate data from the Texas Department of State Health Services, 2009–2011.

## Data Availability

Birth certificate data is restricted and not publicly available. Census tract-level median housing age data were obtained from the American Community Survey, 2007–2011, available at https://www.census.gov/programs-surveys/acs/data.html (accessed on 5 January 2021). Census tract-level poverty data were obtained from the American Community Survey, 2006–2010, available at https://data.census.gov/cedsci/all?q=Income%20and%20Poverty (accessed on 1 January 2021). Rural Urban Continuum Codes, were obtained from the U.S. Department of Agriculture, 2013 at https://www.ers.usda.gov/data-products/rural-urban-continuum-codes.aspx (accessed on 9 December 2021).

## Appendix A

**Table A1:** Odds and 95% confidence intervals of low birth weight, 2009–2011 Texas natality files, *n* = 1,040,642.

Characteristic	Bivariate Models	Demographic Model	Full Model
Child’s sex			
Female	1.17 (1.15–1.19)	1.17 (1.15–1.19)	1.18 (1.16–1.190)
Male	1	1	1
Mother’s age (range 11–57)			
11–19	1.33 (1.30–1.36)	1.01 (0.98–1.03)	0.89 (0.87–0.92)
20–34	1	1	1
35+	1.17 (1.15–1.20)	1.32 (1.29–1.35)	1.40 (1.36–1.43)
Mother’s race/ethnicity/nativity			
Black, U.S.-born	2.36 (2.30–2.42)	2.16 (2.11–2.22)	2.02 (1.96–2.07)
Black, immigrant	1.24 (1.15–1.33)	1.24 (1.15–1.33)	1.19 (1.10–1.29)
Hispanic, U.S.-born	1.32 (1.30–1.35)	1.25 (1.22–1.28)	1.16 (1.13–1.19)
Hispanic, immigrant	0.99 (0.97–1.02)	0.98 (0.95–1.00)	0.82 (0.80–0.85)
White, U.S.-born	1	1	1
White, immigrant	0.85 (0.79–0.91)	0.85 (0.79–0.92)	0.88 (0.81–0.95)
Other, U.S.-born	1.48 (1.38–1.60)	1.46 (1.36–1.58)	1.47 (1.36–1.59)
Other, immigrant	1.34 (1.28–1.40)	1.37 (1.31–1.43)	1.41 (1.36–1.48)
Mother’s marital status			
Unmarried	1.44 (1.42–1.47)	1.25 (1.23–1.28)	1.09 (1.07–1.11)
Married	1	1	1
Parity			
First birth	1	1	1
Second–fourth birth	0.71 (0.70–0.72)	0.73 (0.72–0.75)	0.70 (0.69–0.71)
Fifth+ birth	0.94 (0.91–0.98)	0.91 (0.87–0.94)	0.80 (0.77–0.83)
Mother’s education			
Did not finish high school	1.50 (1.46–1.53)		1.40 (1.35–1.45)
High school graduate	1.46 (1.43–1.50)		1.28 (1.23–1.32)
Some college	1.32 (1.29–1.36)		1.16 (1.13–1.20)
College graduate	1		1
Father’s education			
Missing	2.01 (1.95–2.06)		1.43 (1.38–1.49)
Did not finish high school	1.41 (1.37–1.45)		1.28 (1.23–1.33)
High school graduate	1.48 (1.45–1.53)		1.28 (1.24–1.33)
Some college	1.27 (1.23–1.30)		1.13 (1.10–1.17)
College graduate	1		1
Delayed prenatal care			
Yes	1.13 (1.11–1.15)		1.02 (1.00–1.04)
No	1		1
Urban/rural status			
Urban	0.95 (0.92–0.98)	0.92 (0.89–0.95)	0.97 (0.94–1.00)
Rural	1	1	1
Median housing age + high poverty, tract level			
<1975 and ≥20% poor	1.24 (1.21–1.28)	1.14 (1.11–1.16)	1.09 (1.07–1.11)
1975+ and/or <20% poor	1	1	1
U_0j_		0.018	0.013
-2LL		488,982	463,575.8
AIC		489,016	463,625.8
BIC		489,127.2	463,789.4

Sources: American Community Survey, 2007–2011, U.S. Department of Agriculture Rural Urban Continuum Codes, 2013, and birth certificate. Data from the Texas Department of State Health Services, 2009–2011.

**Table A2:** Odds and 95% confidence intervals of small-for-gestational age, 2009–2011 Texas natality files, *n* = 1,040,642.

Characteristic	Bivariate Models	Demographic Model	Full Model
Child’s sex			
Female	1.60 (1.58–1.62)	1.61 (1.59–1.63)	1.61 (1.59–1.63)
Male	1	1	1
Mother’s age (range 11–57)			
11–19	1.43 (1.41–1.46)	1.09 (1.06–1.11)	0.97 (0.95–0.99)
20–34	1	1	1
35+	0.86 (0.85–0.88)	0.96 (0.94–0.98)	1.01 (0.99–1.03)
Mother’s race/ethnicity/nativity			
Black, U.S.-born	2.19 (2.15–2.23)	2.00 (1.95–2.04)	1.88 (1.83–1.92)
Black, immigrant	1.24 (1.17–1.31)	1.31 (1.24–1.40)	1.23 (1.16–1.31)
Hispanic, U.S.-born	1.32 (1.30–1.34)	1.23 (1.21–1.25)	1.16 (1.14–1.18)
Hispanic, immigrant	1.06 (1.04–1.08)	1.08 (1.06–1.10)	0.92 (0.90–0.94)
White, U.S.-born	1	1	1
White, immigrant	0.94 (0.89–0.99)	0.99 (0.94–1.05)	1.00 (0.94–1.06)
Other, U.S.-born	1.45 (1.37–1.54)	1.43 (1.35–1.52)	1.44 (1.35–1.53)
Other, immigrant	1.82 (1.77–1.88)	1.97 (1.91–2.03)	2.00 (1.93–2.06)
Mother’s marital status			
Unmarried	1.47 (1.45–1.49)	1.26 (1.25–1.28)	1.11 (1.09–1.13)
Married	1	1	1
Parity			
First birth	1	1	1
Second–fourth birth	0.66 (0.66–0.67)	0.71 (0.70–0.72)	0.67 (0.66–0.68)
Fifth+ birth	0.67 (0.66–0.70)	0.73 (0.71–0.76)	0.63 (0.61–0.66)
Mother’s education			
Did not finish high school	1.51 (1.48–1.54)		1.39 (1.35–1.43)
High school graduate	1.48 (1.45–1.51)		1.31 (1.28–1.35)
Some college	1.27 (1.25–1.30)		1.16 (1.13–1.19)
College graduate	1		1
Father’s education			
Missing	1.81 (1.78–1.86)		1.21 (1.18–1.25)
Did not finish high school	1.33 (1.30–1.36)		1.11 (1.08–1.14)
High school graduate	1.38 (1.35–1.41)		1.13 (1.10–1.16)
Some college	1.17 (1.15–1.20)		1.04 (1.01–1.07)
College graduate	1		1
Delayed prenatal care			
Yes	1.32 (1.30–1.33)		1.21 (1.19–1.22)
No	1		1
Urban/rural status			
Urban	0.92 (0.89–0.94)	0.89 (0.87–0.91)	0.92 (0.90–0.94)
Rural	1	1	1
Median housing age + high poverty, tract level			
<1975 and ≥20% poor	1.22 (1.20–1.25)	1.11 (1.09–1.13)	1.07 (1.05–1.09)
1975+ and/or <20% poor	1	1	1
U_0j_		0.015	0.012
-2LL		708,798.7	674,979.4
AIC		708,832.7	675,029.4
BIC		708,943.9	675,193

Sources: American Community Survey, 2007–2011, U.S. Department of Agriculture Rural Urban Continuum Codes, 2013, and birth. Certificate data from the Texas Department of State Health Services, 2009–2011.
